# Bœnninghausen’s Croup Powders

**Published:** 1889-07

**Authors:** A. McNeil


### BŒNNINGHAUSEN'S CROUP POWDERS.

"I appeal from Philip drunk to Philip sober." I appeal from Dr. Wells as the apologist of doubtful practice to P. P. Wells, the valiant defender of true Homœopathy. In my first paper criticizing Dr. Wells's advocacy of Bœnninghausen's croup powders, I stated that I had received the impression that they were intended for domestic use. I will now quote from Bœnninghausen's *Aphorismen des Hippokrates*, page 403: "It is, therefore, advisable to keep the remedies always on hand, as they are always the same, *do not deteriorate in keeping,* and are for the *commencement always the same,* and more particularly because the disease (croup), is accustomed to make its appearance usually in the *evening,* late or in the middle of the night, when delay and loss of time are unavoidable. And it may be added, the remedies prepared in the most appropriate smallest doses can *never do any harm* if at first the true croup is not present." In the next paragraph he mentions thirteen other remedies that may be indicated in neglected cases. In further confirmation I will quote Carroll Dunham, *American Homœopathic Review,* vol. III, page 536: "It is well known that Dr. Bœnninghausen does not visit his cases, but prescribes chiefly in his office." I admitted that, at that time, Aconite being the epidemic remedy, it was indicated in the beginning of all cases of croup. But it does not follow that it is *the* remedy now, any more than because Hahnemann cured a great many cases of typhus fever in 1813 with Bryonia and Rhus they are to be given in all cases of typhus at the present, and, moreover, it doesn't follow that, because for *domestic use,* Aconite, Hepar, and Spongia were recommended that physicians should give nothing else.

The venerable Doctor says, "There are names of sickness which, when spoken, present to the mind a picture which is so perfectly repeated in the successive examples of this sickness, that the name contains in it, to the intelligent mind, a more or less complete expression of the totality of the phenomena of that sickness." Let me place next to this a quotation of that staunch defender of true Homœopathy, Adolph Lippe, *American Homœopathic Review*, vol. III, page 148: "The true physician will never be guided by the name of the disease or by the pathological condition of the diseased organ in the choice of the remedy." And, on the next page, "no one familiar with Homœopathy can believe in specific medicines for specific diseases."

It might be embarrassing to the venerable defender of pure Homœopathy to employ the same phrases used by the polypaths, for that is the way they justify their unhomœopathic practices.

I will now describe cases of croup which I have cured by other remedies than are in Bœnninghausen's powders, and ask your venerable correspondent how he would treat them. The little patient lies perfectly still, owing to the pains caused by the slightest movement. He breathes very superficially and restrains the desire to cough as long as possible, and cries or at least distorts his face when he can no longer avoid it. Or a case in which the patient changes his position constantly, not from mental anxiety but because movement gives temporary relief, and, after the case has continued long enough for their development, herpes cover the lips; or when the saliva runs constantly from his mouth, or, if old enough, spits all the time. I might go on and describe other cases I have seen to which I gave Lachesis or Tartar emetic. In all of these cases the diagnosis of membraneous croup was clear. But, for fear my testimony may be impugned, I will give the evidence of one whom Dr. Wells will not presume to deny. (See Guernsey's *Obstetrics*, third edition, page 816): "Chamomilla; with this unusual remedy I once cured a very bad case of croup, when all other medicines had failed to afford relief, and I despaired of the child's life, from observing very strongly marked in this case that characteristic symptom of Chamomilla, the child must be carried up and down the room for relief. I was led to give this remedy, which, much to my delight, was followed by speedy recovery." It is a fair presumption that Prof. G. had given Aconite, Hepar, and Spongia.

I ask your venerable correspondent if in the above-mentioned cases he would give Bœnninghausen's powders? I have too high an opinion of his skill and knowledge of Homœopathy to think he would. And I am of opinion that none of the four hundred cases cured were such as I have delineated or like the one mentioned by Guernsey, or they would not have all recovered. Hahnemann says, "Every time that Aconite is chosen as a homœopathic remedy it is especially necessary to regard the moral (mental) symptoms, and be careful that they resemble those which belong to it." I have seen many cases of croup which did not have mental anxiety and restlessness which, Hahnemann says, is necessary to justify the administration of Aconite.

For the information of myself and others, will Dr. Wells be kind enough to give us the names of those diseases which are exceptions to Hahnemann's instructions that we must not prescribe for the names of diseases? as in a careful study of the works of Hahnemann I have never seen any mentioned.

I will now proceed to my second point, viz. : It (giving Bœnninghausen's powders) is an alternation of remedies.

I will quote Dunham as to the definition of alternation and succession of remedies, as the venerable Doctor says that "I am intelligent enough to recognize the difference between *succession* and 'alternation.'" *American Homœopathic Review*, vol. III, p. 530 : "*Alternation* and *succession* of remedies are not generally understood to be identical processes. By alternation we think practitioners generally understand the prescription at one and the same time of two or more remedies to follow each other at short intervals, the symptoms of these remedies taken *altogether* being thought to cover the symptoms of the case more completely than those of either remedy *alone* would do. The prescription is the result of one single examination of the patient and of one single comparison of the symptoms with the materia medica.

"But when a succession of remedies is given in either an acute or chronic disease, the understanding is that the first remedy, having been carefully selected, is allowed to exhaust its action alone, and then a collection of the symptoms which the patient *then* presents is made, and the case is prescribed for *afresh*, almost as if it were a new case, and this process is repeated, each new prescription being the subject of a new study until the case is cured. Such a process is equivalent to prescribing for a number of new and independent cases, and it is evidently not incompatible with the theory of a true homœopathic prescription."

I acknowledge that further on in that paper Dunham attempts to prove that the administration of Bœnninghausen's powders is "successive," but, with all my respect for him, I am of opinion that he makes a miserable failure of it. I ask Dr. Wells to make use of these definitions in deciding on whether or not such prescribing is alternation.

In regard to my third point, viz., "It is not the most successful way of curing croup."

The Doctor asks if I have a more successful record to present of curing croup. No, I have not so large a number—perhaps fifty without a death in Hahnemann's way—while with Bœnninghausen's I have had several fatal results. But, Yankee-like, let me ask the Doctor another question: Hahnemann cured one hundred and eighty-seven cases of typhus with Rhus and Bryonia without a death; has the Doctor a better way? I will give an answer he has already written in that monograph on "Typhoid Fever," which will carry his name down to the latest posterity. I quote from the *American Homœopathic Review*, vol. III, page 391: "We are not to alternate remedies * * * and are to neither give nor to alternate Bry. and Rhus because it is typhoid fever." But he now says we are to give Aconite, Hepar, and Spongia because it is croup. I ask your valued correspondent if that is not making a fine distinction, and I am willing that the many true Hahnemannians who read THE HOMŒOPATHIC PHYSICIAN shall decide whether or not Dr. Wells is consistent.

I owe much to your veteran correspondent for the many noble defenses he has made of true Homœopathy. His monographs on diarrhœa, dysentery, scarlet fever, typhoid fever, and other papers, with those of the genial Carroll Dunham, guided my erring footsteps into that path in which Hahnemann led. And I feel almost guilty of presumption in thus pointing out what I believe is not in accordance with the precepts which I have learned from him. And I would not have done it at all but for my failure with Bœnninghausen's powders in saving sweet young lives from going out in death, and which I believe might and ought to have been saved. And that I have since found a surer way, Hahnemann's way.

A. McNEIL.

